# The effects of adolescent social isolation and group housing on adolescent binge drinking in mice

**DOI:** 10.1371/journal.pone.0354475

**Published:** 2026-07-24

**Authors:** Jyoti Lodha, Alanna Morgan, Rithika Balguri, Vera Purcell, Jennifer T. Wolstenholme

**Affiliations:** 1 Pharmacology and Toxicology Department, Virginia Commonwealth University, Richmond, Virginia, United States of America; 2 Alcohol Research Center, Virginia Commonwealth University, Richmond, Virginia, United States of America; Methodist University Cape Fear Valley Health School of Medicine, UNITED STATES OF AMERICA

## Abstract

Adolescence is a developmental period characterized by behavioral changes such as sensation seeking, impulsivity, and diminished self-control to inhibit behaviors, which can lead to increased risky behavior, including the initiation of binge drinking. Due to the necessity of peer-peer social interactions for proper development, social isolation can be particularly disruptive at this time, altering proper neural development and function and increasing risk for alcohol misuse. To investigate the effects of social context on ethanol drinking, we modulated the social environment of a mouse using single housing, neighbor cages, or group housing and evaluated ethanol drinking behavior in the intermittent 2-bottle choice and drinking in the dark models. Using neighbor cages versus social isolation, ethanol intake was largely unaffected by housing condition from adolescence into early adulthood. However, single housing increased adolescent ethanol intake using a drinking in the dark model in both sexes. Group housed mice with concurrent ethanol access drank significantly less than isolated animals. Manipulating the number of ethanol bottles per cage increased the volume of ethanol consumed, but did not affect the relative differences in intake between group and single housed mice. Adolescent binge drinking increased adult intake only in group housed mice. In sum, social isolation during adolescence increased ethanol intake while drinking with conspecifics decreased ethanol intake. Drinking in isolation tended to increase ethanol intake, suggesting the presence of a conspecific alters the motivation to drink ethanol.

## Introduction

Adolescence is a period of increased peer-peer interaction and risk-taking behavior. During this developmental period, defined as age 12–20 in humans and ~postnatal day (P) 21–60 in rodents, both human and rodent brains undergo significant synaptic pruning and increased myelination [1, 2]. Alcohol is the most commonly misused intoxicant among adolescents with 7.1 million underage youth (age 12–20) reporting drinking alcohol in the past month. Many of these youth (4.3 million) did so in the form of binge drinking, defined as four or more drinks for women or five or more drinks for men in a single occasion [3]. Binge drinking can be particularly harmful in adolescence as alcohol may delay or disrupt critical ongoing neurodevelopment, with profound consequences in adult behavior, brain structure, connection, and function [4, 5]. In adolescent drinkers, any alcohol use was associated with accelerated grey matter reductions in frontal and cingulate cortices and subcortical regions involved in reward and memory [6]. In preclinical models, adolescents respond differently to alcohol than adults, showing increased sensitivity to alcohol’s rewarding aspects and decreased sensitivity to the aversive aspects of alcohol [7]. This may enable adolescents to increase their alcohol consumption, giving rise to the binge drinking behavior commonly seen among this age group, while subsequently increasing the risk for developing an alcohol use disorder (AUD) later in life [8]. The risk of developing adult AUD increases the younger one first engages in alcohol consumption [9, 10]. In a recent longitudinal study excluding other risk factors, adolescent and young adult solitary alcohol use was associated with increased adult AUD symptoms, particularly in females [11], highlighting the importance of understanding the combined role of social isolation and binge drinking in adolescence.

Social behavior neurocircuitry may be uniquely altered by alcohol’s actions in adolescence. Likewise, exposure to alcohol in adolescence can alter social behaviors. Social play behavior is modulated through some of the same signaling pathways that mediate reward, such as the prefrontal cortex, nucleus accumbens, and striatum [12]. Illicit drugs also alter the expression of play behavior [13]. In response to low doses of ethanol, juvenile rats showed social facilitation and were more sensitive to ethanol’s rewarding effects than adults, increasing the incentive value for adolescents [13, 14]. Alternatively, adolescent rats were less sensitive than adults to the social impairing, motor disrupting, aversive, and sedative effects of higher doses of ethanol [15, 16]. These opposing effects of ethanol likely serve to temper drinking in adult rodents in comparison to adolescents. Indeed, adolescent rodents drink more ethanol than their adult counterparts [17–21]. These effects appear to be limited to adolescence, as increased ethanol preference does not occur in isolated adult rodents [22–24], suggesting that early timing of exposure to social isolation is crucial in order to produce significant changes in the sensitivity to ethanol later in adult life.

Early life stressors are associated with alcohol misuse in underage populations. Among heavy drinking college students, drinking alone was associated with increased feelings of loneliness and a tendency to drink as a means of coping with stress [25, 26]. Individuals who report feelings of social isolation, decreased social support, and increased Alcohol Use Disorder Identification Test (AUDIT) scores exhibited altered connectivity patterns between the hypothalamus and ventral striatum, which also correlated with a metric of perceived burdensomeness [25, 27]. Drinking alone at an early age may be predictive of an AUD at age 35, particularly among females [11]. Lower resilience to stress (i.e., heightened aggression or emotional instability) correlated with increased risk of addictive substances, including increased risk of alcohol consumption and dependence [28]. In the few studies that directly investigated drinking behavior in single versus group housed rodents, post-weaning social isolation usually increased ethanol consumption in adolescent rats and mice [23, 29, 30], but did not always alter ethanol consumption [31]. When a partial barrier system was used, housing in groups or in isolation did not always alter drinking in adult rodents and was sex dependent [32, 33]. Thus, there is a subpopulation of individuals who engage in problematic drinking when they are alone and this is understudied in adolescent models.

While studies on adolescent social isolation and ethanol drinking have investigated how either factor affects adult ethanol consumption, few studies have addressed what occurs when both are incurred simultaneously during adolescence. The aim of these studies was to primarily investigate how social context in adolescence affects adolescent ethanol intake in C57BL/6J mice. We also examined how adolescent social context and history of ethanol drinking altered adult drinking. We found that social setting had a strong influence on ethanol intake, both in adolescents and later in adulthood. Mice that drank alone, in a single housed setting, tended to consume the most amount of ethanol. However, the presence of a conspecific in the same cage reduced drinking. When ethanol access occurred in the proximity, but not the same cage with another mouse, ethanol intake was only slightly decreased in comparison to the single housed condition. Binge drinking in adolescence increased adult intake and preference, but only in group housed social drinking mice on select days.

## Materials and methods

### Animals

In all experiments, male and female C57BL/6J mice from Jackson Laboratory (Bar Harbor, ME, USA) arrived at post-natal day (P) 21–22 and were housed in same-sexed cages (4/cage) for ~1 week in an American Association for Laboratory Animal Care (AALAC)-accredited facility under 12-h light/dark cycles. Mice were given access to food and water*ad libitium*, with the exception of the four-hour Drinking in the Dark (DID) sessions where diluted ethanol was the only liquid offered. Mice were randomly assigned to each treatment and housing condition. Mice were either housed in standard shoebox cages or in neighbor housed complexes with SaniChip (Teklad #7090) bedding. The neighbor housing complexes are four standard mouse cages connected by circular ports (6 cm in diameter) with a wire mesh (8 mm welded metal mesh with 1 cm^2^ wide openings) within the port [34, 35]. These cages allow animals to live individually in their own cages, but allow limited interaction with the other mice in the complex. Each neighbor complex had 4 mice – one in each of the four connected cages. Single housed mice were housed 1 mouse per cage, while group housed mice were housed 4 mice per standard shoebox cage. Each cage (single, neighbor or group) was given a single cotton 1-inch square in SaniChip bedding. All animal housing and care was conducted with the approval of the Virginia Commonwealth University Institutional Animal Care & Use Committee (IACUC) and in accordance with the NIH Guide for the Care and Use of Laboratory Animals Council [36].

### Experiment 1: 2BC intermittent access in single and neighbor housed mice

Two-bottle choice (2BC) intermittent ethanol access has been shown to escalate drinking behavior in rodents [37]. On P26, adolescent male or female C57BL/6J mice were single (1/cage) or neighbor housed (4/complex) for the 2BC intermittent access studies. We have previously shown that neighbor housing mitigates cognitive deficits incurred by social isolation during adolescence [35]. Immediately prior to the onset of the dark cycle, mice were given one bottle of 15% (w/v) ethanol and one bottle of tap water (2BC). From P28-P57, mice had free access to the drinking bottles on Mondays, Wednesdays, and Fridays for 24 hours, after which the volume of each bottle was recorded and two bottles were replaced with a single water bottle. The position of the ethanol and water bottles was alternated each drinking session. Mice had three 24-hr drinking sessions each week for four weeks.

### Experiment 2: Single, neighbor, and group housed Drinking in the Dark (DID)

The purpose of this experiment was to test how a larger living area and increased access to drinking bottles in the neighbor housing cages affected ethanol intake in comparison to single or group housed social drinkers. We used a modified DID drinking model to mirror adolescent binge drinking in humans – limited access while still reaching a blood ethanol content (BEC) >80 mg/dL [38, 39]. This model typically allows mice to reach blood ethanol levels greater than 80 mg/dL [38] and has been used to show increases in adult drinking after ethanol exposure in adolescence [39, 40]. Adolescent male C57BL/6J mice were organized into three groups: single (1/cage), neighbor housed (4/complex), or group housed (4/cage) beginning on P27. The same neighbor complexes from Experiment 1 were used; however, the wire mesh was removed from the ports, allowing all four mice to freely move about the four interconnected cages and interact with one another. Three hours into the dark cycle, the standard water bottle was removed from each cage and mice were given access to a single bottle of 20% (v/v) ethanol for four hours. Each neighbor complex had one ethanol drinking bottle in each living space; group housed social mice had access to one bottle per cage. Mice underwent four drinking sessions/week for three weeks (P27-P44). Due to the large number of housing conditions in this experiment and the capacity of the specialized neighbor cages, only males were used in Experiment 2.

### Experiment 3: Single and group housed DID in individual or social drinking cages

To compare how drinking in isolation versus drinking in a social setting altered binge ethanol intake, three drinking situations were assessed. Beginning on P27, adolescent male and female C57BL/6J mice were single housed (1/cage), group housed (4/cage) and isolated for drinking sessions, or group housed social (4/cage) and remained in groups during the drinking sessions. All animals were moved to drinking cages 2.5 hours prior to the drinking session. The single and group housed mice were separated into individual drinking cages. The group housed social drinkers were placed into a single drinking cage to drink together with one ethanol bottle. Drinking cages remained the same throughout each week. Three hours into the dark cycle, mice were given access to a single bottle of 20% (v/v) ethanol for four hours and then returned to their assigned home cages. Mice underwent four drinking sessions/week for three weeks during adolescence from P27-P44. Retro-orbital eye bleeds were conducted on P44 immediately following the drinking session to assess BECs.

### Experiment 4: Single and group housed social DID in home cages with a variable number of ethanol bottles

Since bottle access could be limiting ethanol intake in a group housed setting, this experiment varied the number and type of bottles offered to group housed social or single housed mice. Adolescent C57BL/6J males were single (1/cage) or group housed social (4/cage) beginning on P27. All mice remained in their home cages during the drinking sessions. Three hours into the dark cycle, mice were given access to either: (a) 2 bottles of 20% (v/v) ethanol, 2 bottles of tap water (4BC), (b) 4 bottles of 20% (v/v) ethanol (4Ethanol), or (c) 1 bottle of 20% (v/v) ethanol (1Ethanol). Mice underwent four drinking sessions/week for three weeks during adolescence from P27-P44. Due to the large number of experimental conditions, males were used in Experiments 4 and 5.

### Experiment 5: Single and group housed social 2BC in adulthood after adolescent DID

Experiment 5 utilized the same DID paradigm as Experiments 2–4 in adolescents and the same 2BC paradigm as Experiment 1 in adults. Adolescent male C57BL/6J mice were single (1/cage) or group housed social (4/cage) beginning on P27. Mice were given access to 20% (v/v) ethanol DID or water for four drinking sessions/week (4 hours/day, 3 hours into the dark cycle) for three weeks during adolescence from P27-P44. In order to assess ethanol intake, preference, and potential escalation of drinking in adulthood, all mice (ethanol DID and water groups) were single housed and underwent a 2BC procedure from P60-P79. Three hours into the dark cycle, mice were given access to a single bottle of water and a single bottle of 20% (v/v) ethanol on Mondays, Wednesdays, and Fridays. Thus, adolescent mice had 4 days of ethanol access per week in the DID sessions and as adults had 3 days of ethanol access in the intermittent 2BC paradigm. All animals remained in their home cage during the drinking sessions. The position of the ethanol and water bottles was alternated each drinking session in the intermittent 2BC studies.

### Intake and preference calculations

Our ethanol and water bottles are constructed from 10mL graduated serological pipettes fitted with a sipper tube and rubber stopper allowing us to accurately record the volume displaced during the drinking sessions. Individual intake was calculated as grams of ethanol consumed per kilogram body weight of each animal. Ethanol preference was calculated as the volume of ethanol divided by the total fluid (ethanol + water) volume. For the group housed social drinkers, whose individual intake cannot be obtained, we calculated the grams ethanol consumed by the cage divided by total weight of the cage [41]. In Experiment 5, total fluid for group housed mice was calculated as total volume consumed in 24 hours divided by 4 to account for the four mice in the cage. In all experiments, a separate set of bottles was placed on empty cages and used to account for evaporation of ethanol and water during the drinking sessions.

### Blood Ethanol Concentration (BEC)

Animals were briefly anesthetized with isoflurane and blood was collected via retro-orbital eye bleeds. Whole blood was stored in BD Microtainer EDTA tubes at -80^o^C until samples were analyzed for blood ethanol concentration. BECs were analyzed on a GC Headspace (courtesy of Dr. Keith Shelton) using 1-propanol as an internal standard [42].

### Statistical analysis

Data was analyzed in GraphPad Prism Version 10.0.2 (GraphPad Software San Diego, CA). Ethanol intake and preference data was analyzed using two-way repeated measures ANOVA (housing x day) or mixed model repeated measures ANOVA (housing x adolescent treatment x day) followed by Tukey’s post-hoc tests. We are most interested in how social experience alters adolescent ethanol drinking behavior within each sex. Female rodents are known to consume more ethanol than males. Thus, specific differences in ethanol drinking between males and females is not a major focus of these studies. Males and females were analyzed separately due to our *a priori* hypothesis that males and females will drink differently. The effect size for all experiments was calculated based on ethanol intake data in G*Power Version 3.1.9.6 (G*Power Heinrich Heine University, Düsseldorf Germany). A value of p < 0.05 was considered a significant difference. Data are presented as mean + /- SEM.

## Results

### Experiment 1: 2BC ethanol intake and preference are not altered by neighbor housing in adolescent males and females

**Fig 1:**
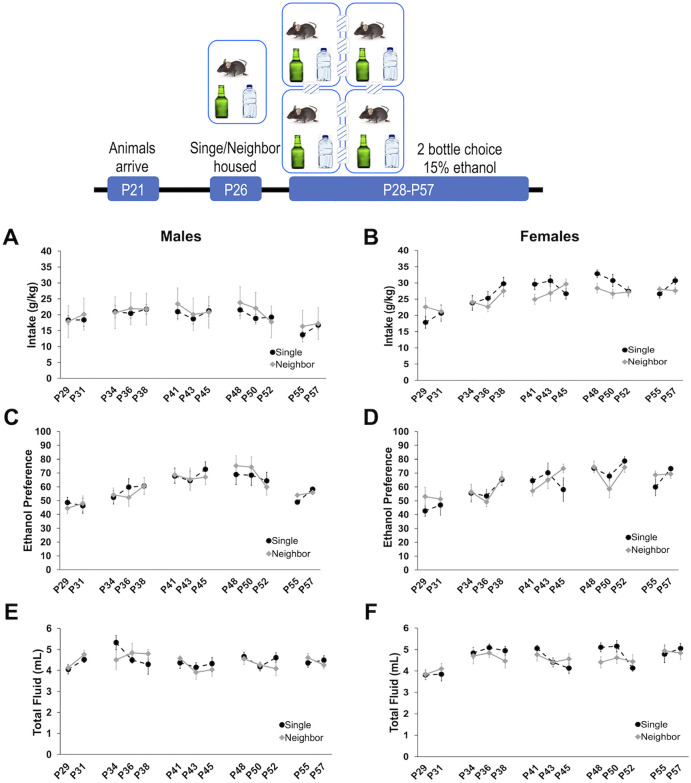
Intermittent ethanol access in single and neighbor housed mice does not alter ethanol intake, preference, or total fluid consumption in males or females using a 2-bottle choice paradigm. Ethanol intake in males (A) and females (B) is not affected by housing when comparing single and neighbor housed mice in adolescence. Intake increases over time in both sexes. Preference and total fluid in males (C, E) and females (D, F) is not affected by housing when comparing single and neighbor housed mice in adolescence. n = 12/group.

Single and neighbor housed male and female adolescent mice were given 24-hr intermittent access to 2BC ethanol from P29-P57 (Fig 1). Two-way repeated measures ANOVA with housing and day as factors were performed separately for males and females. In males, housing did not alter ethanol intake (Fig 1A, F_housing_ (1, 22) = 0.6703, p = 0.4217). Ethanol intake increased over time (F_day_ (12, 258) = 4.480, p < 0.0001). There was no significant interaction between housing and day to report in males (F_interaction_ (12, 258) = 0.5103, p = 0.9073). In females, ethanol intake was not affected by housing (Fig 1B, F_housing_ (1, 22) = 0.8352, p = 0.3707), but intake increased over time (F_day_ (12, 255) = 8.212 p < 0.0001). There was a significant housing x day interaction for intake in females (F_interaction_ (12, 258) = 1.888, p = 0.0361), where ethanol intake increased over the course of the study (from P29-P34 to P45-P50) within each housing group. Despite there being no significant differences between housing conditions, power analysis estimates are greater than 98% for males and females.

Ethanol preference was not affected by housing in males (Fig 1C, F_housing_ (1, 22) = 0.00102, p = 0.9748); but there was a statistically significant effect of day (F_day_ (12, 262) = 7.247, p < 0.001). There was no significant interaction between housing and day for ethanol preference in males (F_interaction_ (12, 262) = 0.446, p = 0.9433). There was no effect of housing on female ethanol preference (Fig 1D, F_housing_ (1, 22) = 0.028, p = 0.8687), but a statistically significant effect of day was found with a general trend to increase over time (F_day_ (12, 261) = 8.819, p < 0.0001). There was no significant interaction between housing and day for female ethanol preference (F_interaction_ (12, 261) = 1.459, p = 0.1398).

Total fluid did not differ due to housing in males (Fig 1E, F_housing_ (1, 22) = 0.0415, p = 0.8405), but increased over time (F_day_ (12, 263) = 2.166, p = 0.0137). There was no significant interaction in male total fluid (F_interaction_ (12, 263) = 1.210, p = 0.2761). In females, total fluid did not differ due to housing (Fig 1F, F_housing_ (1, 22) = 0.210, p = 0.6513), but increased over time (F_day_ (12, 261) = 7.528, p < 0.0001). There was no significant interaction in female total fluid (F_interaction_ (12, 261) = 1.462, p = 0.1385).

### Experiment 2: Social isolation increases adolescent DID

**Fig 2:**
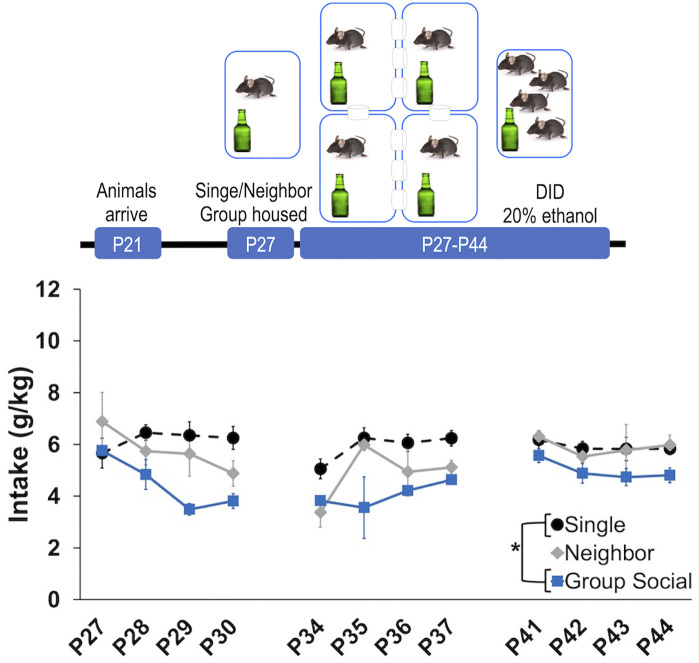
Adolescent housing condition alters ethanol intake in the Drinking in the Dark (DID) paradigm in male mice. Single and neighbor housing increases ethanol intake as compared to group housed social drinkers. * p < 0.05 by two-way repeated measures ANOVA. n = 12 per group for single; n = 3/group for neighbor; n = 4/group for group housed social.

In Experiment 2, and onward, we shifted to a modified DID paradigm as this model more closely mirrors human adolescent binge drinking. In this experiment, the wire mesh was removed from the ports in the neighbor complexes to allow the four mice to freely roam among the four interconnected cages and we added a group housed social drinking cohort (4/cage), in which mice live and drink together in the same cage. As seen in Fig 2, there was a significant effect of housing (F_housing_ (2, 16) = 10.97, p = 0.0010) and day (F_day_ (11, 171) = 2.613, p = 0.0042). Tukey’s post-hoc analysis revealed that single housed males drank significantly more than group housed social males (p = 0.0007), with a large effect size (0.79) at greater than 99% power. There was a trend towards a difference between neighbor and group housed social mice (p = 0.0717), which is likely driven by transient increases in intake by neighbor mice on P29 and P35. Based on post-hoc analysis, there were no significant differences between neighbor and single housed mice (p = 0.3735). There were no significant interactions between housing and day to report (F_interaction_ (22, 171) = 1.145, p = 0.3044).

### Experiment 3: Drinking alone increases ethanol intake compared to drinking together

**Fig 3:**
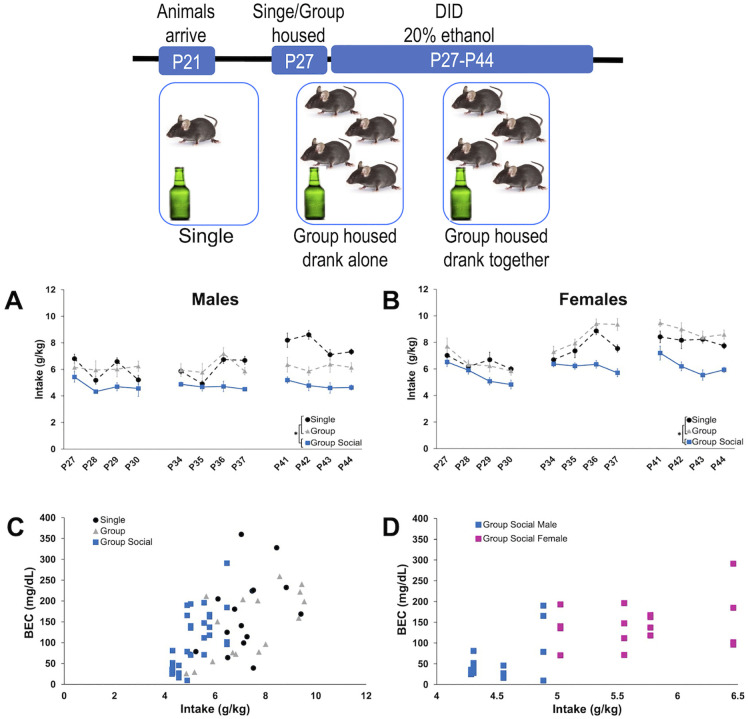
Group housed social drinking decreases DID ethanol intake in males and females in adolescence. Single and group housed males (A) and females (B) drank significantly more than their group housed social drinking counterparts throughout adolescence (P27-P44). (C) On P44, blood ethanol concentrations (BECs) were significantly correlated to ethanol intake (R^2^ = 0.6304, p < 0.0001). (D) Ethanol intake was detectable within each cage in both males and females. * p < 0.05 by two-way repeated measures ANOVA, n = 8/group for single & group; n = 4/group for group housed social.

Since single housed males drank more than group housed social mice, but ethanol intake in neighbor housed mice did not differ from the other groups in Experiment 2, we designed this experiment to directly ask whether intermittent separation into single housing would mimic group housed social drinkers’ intake while still allowing us to assess individual ethanol intake. Male and female adolescents were exposed to single housing, group housing with the ability to drink in a social setting, or group housing with drinking sessions in isolated drinking cages. There was a significant effect of housing (Fig 3A, F_housing_ (2, 17) = 7.679, p = 0.0042) and day (F_day_ (11, 187) = 2.029, p = 0.0278) and a trend towards an interaction between housing and day on ethanol intake in males (F_interaction_ (22, 187) = 1.559, p = 0.060). Single and group housed males in individual cages drank significantly more than group housed social males. The effect size value, 1.12, was large and power was greater than 99%. A similar trend was observed in females with significant effects of housing (Fig 3B, F_housing_ (2, 17) = 21.58, p < 0.0001), day (F_day_ (11, 187) = 12.39, p < 0.0001), and an interaction of housing and day (F_interaction_ (22, 187) = 1.767, p = 0.0228). Similar to the males, single and group housed females drank significantly more ethanol than group housed social females. The effect size value (0.76) suggests a moderate to high effect of housing, and power was greater than 99%. BECs from whole blood collected on P44 were significantly correlated to ethanol intake after the final drinking session across all groups (Fig 3C, R^2^ = 0.6304, p < 0.0001). BECs were detectable in each group housed animal (Fig 3D).

### Experiment 4: Bottle access does not affect adolescent intake; socially isolated mice consistently drink more than group housed mice

**Fig 4:**
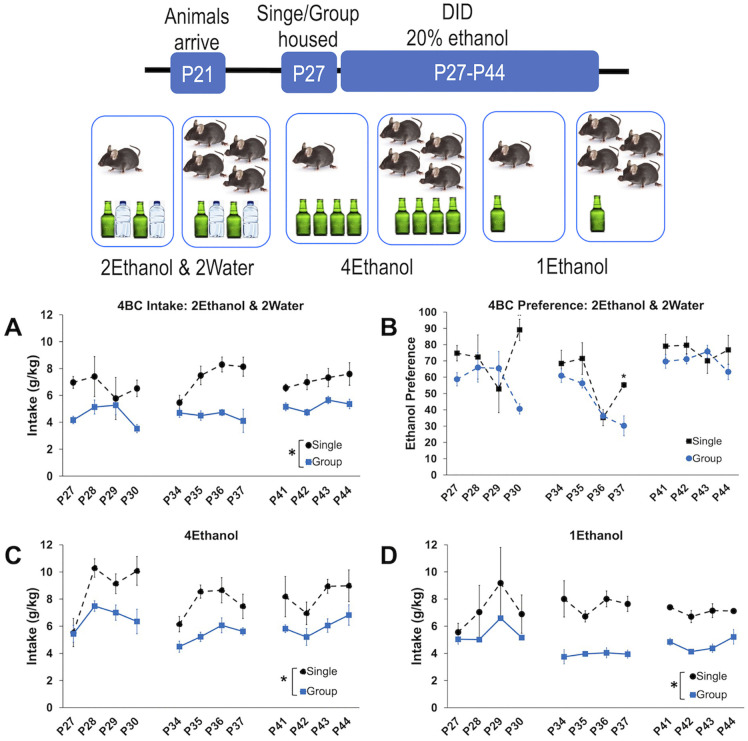
Variation in number of ethanol bottles per cage does not alter the relative intake between single and group housed social drinking males. When given ethanol during adolescence (P27-P44), single housed mice drank more than group housed mice regardless of being given 4BC (A), 4Ethanol (C), or 1Ethanol (D). (B) Ethanol preference was transiently increased in single housed males on P30 and P37. * p < 0.05 by two-way repeated measures ANOVA, n = 8/group for single 4BC & 4Ethanol; n = 6/group for group housed social 4BC & 4Ethanol; n = 3 for 1Ethanol group social & single housed.

Since Experiment 3 showed that group housed social drinking decreased ethanol intake relative to drinking in isolation, our next question was if bottle access could be driving this decreased intake. Although overt dominance behaviors are not characteristic in male mice in this age range, there could still be competition for a single bottle among the four mice [43, 44]. To test this, each cage was given varying numbers of 20% (v/v) ethanol and/or water: 2 water bottles/2 ethanol bottles (4BC), 4 ethanol bottles (4Ethanol), or 1 ethanol bottle (1Ethanol). Due to the number of conditions in this experiment, only males were used. All mice remained in their home cages to reduce any potential stress-induced drinking related to the use of new drinking cages during the DID sessions. Separate two-way repeated measures ANOVAs were performed for each condition with housing and day as factors. In the 4BC group, there was a significant effect of housing on ethanol intake (Fig 4A, F_housing_ (1,11) = 45.30, p < 0.0001), where single housed mice consumed more ethanol than group housed social. The effect size for housing, 1.54, was large and power was greater than 99%. There was no significant effect of day (F_day_ (11, 117) = 1.146, p = 0.3327) or interaction between housing and day (F_interaction_ (11, 117) = 1.199, p = 0.2956) on ethanol intake in the 4BC groups. There was a significant effect of housing (F_housing_ (1, 11) = 6.806, p = 0.0243), and day (F_day_ (11, 117) = 6.271, p < 0.0001) on ethanol preference in the 4BC groups. There was a significant interaction of housing x day on ethanol preference (Fig 4B, F_interaction_ (1, 10) = 2.325, p = 0.0088), with transient increases in single housed ethanol preference at the end of the first two weeks of drinking (on P30 and P37) as compared to group housed social males. In the 4Ethanol group, there was a significant effect of housing (Fig 4C, F_housing_ (1, 10) = 18.92, p = 0.0014) and day (F_day_ (11, 110) = 5.174, p < 0.0001), with isolated mice consuming more ethanol than group housed social. The effect size value for housing was 0.97 and power was greater than 99%. There was no interaction between housing and day in the 4Ethanol group (F_interaction_ (11, 110) = 0.9431, p = 0.5025). For the 1Ethanol group, there was a significant effect of housing (Fig 4D, F_housing_ (1, 4) = 12.41, p = 0.0244) and a trend towards an effect of day (F_day_ (11, 44) = 1.827, p = 0.0780), but no interaction (F_interaction_ (11, 44) = 1.070, p = 0.4060). The effect size value for housing, 1.54, was large and power was greater than 99%. As observed in the previous DID experiments, single housed mice drank significantly more than group housed social mice regardless of the number of ethanol bottles available in the cage.

### Experiment 5: Single housing increases ethanol intake in adolescence and adulthood, while a history of binge ethanol in adolescence increases intake in group housed males in adulthood

**Fig 5:**
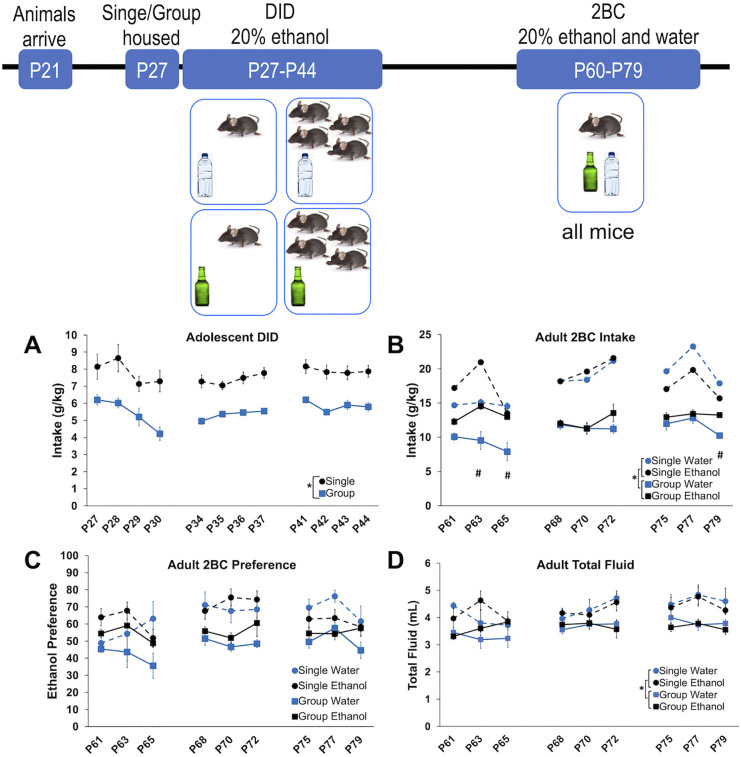
Binge ethanol in adolescence increases adult intake in group housed, but not single housed males. (A) In adolescence, single housed males drank significantly more ethanol than group housed social males. (B) In adulthood, single housed animals, regardless of ethanol history, drank more ethanol in a two-bottle choice (2BC) paradigm than group housed social drinking mice. A prior history of ethanol transiently increased intake in group housed social mice on P63, 65, and 79 compared to ethanol naïve group housed social mice. (C) Ethanol preference was not affected by prior ethanol exposure or housing. (D) Total fluid was higher in single housed mice as compared to group housed social, regardless of a history of ethanol. * p < 0.05 by two-way repeated measures ANOVA (A) or three-way repeated measures ANOVA (B-D). ^#^p < 0.05 significance based on post-hoc test from a two-way repeated measures ANOVA group housed social ethanol vs. water. n = 12/group for all groups in adolescence (A); n = 6/group in adulthood (B-D).

In Experiment 5, male mice were single housed or group housed social and given four hours access to 20% (v/v) ethanol DID or water three hours into the dark cycle (Fig 5A). Single housed mice drank significantly more than group housed social mice during adolescence with a significant effect of housing (Fig 5A, F_housing_ (1, 22) = 55.92, p < 0.0001) and a significant effect of day (F_day_ (11, 242) = 3.955, p < 0.0028). The effect size value for housing, 2.08, was large and power was greater than 99%. There were no significant interactions between housing and day (F_interaction_ (11, 242) = 0.6005, p = 0.8276).

As adults, mice underwent 2BC drinking, as described in Experiment 1. Three-way repeated measures ANOVA revealed a significant main effect of housing (Fig 5B, F_housing_ (1, 19) = 42.66, p < 0.0001) with single housed mice drinking more than group housed social mice. There was a significant main effect of day (F_day_ (8,152) = 6.437, p < 0.001), a day x housing interaction (F_interaction_ (8, 152) = 2.094, p = 0.0396), and a day x housing x adolescent treatment interaction (F_interaction_ (8, 152) = 2.735, p = 0.0076). The effect size value for housing, 1.61, was large and power was greater than 99%. To further dissociate the interactions, we ran two-way repeated measures ANOVAs for each housing condition separately for ethanol intake in adulthood. In group housed social mice, there was a significant effect of adolescent treatment on ethanol intake (F_treatment_ (1, 10) = 8.760, p = 0.0143), with group housed social ethanol mice drinking more than group housed social water mice. There was an interaction between day and adolescent treatment (F_interaction_ (8, 80) = 2.178, p = 0.0378). Based on post-hoc tests, this interaction was driven by days P63, P65, and P79, where group housed social ethanol mice drank more than group housed social water mice (p = 0.0007, p = 0.006, p = 0.0399, respectively). There was no significant effect of day in group housed social mice (F_day_ (8, 80) = 1.478, p = 0.1783). In single housed mice, there was no significant effect of adolescent treatment (F_treatment_ (1, 9) = 0.0022, p = 0.9640), nor a day x adolescent treatment interaction (F_interaction_ (8, 72) = 1.813, p = 0.0885). A significant main effect of day (F_day_ (8, 72) = 5.074, p < 0.0001) was found which is reflected in the increased intake over time in the single housed mice.

Regarding ethanol preference in adulthood, a three-way repeated measures ANOVA revealed a significant effect of day (Fig 5C, F_day_ (8,152) = 2.816, p = 0.0061) and housing (F_housing_ (1, 19) = 15.82, p = 0.0008) but no effect of adolescent treatment (F_treatment_ (1, 19) = 1.247, p = 0.2780), day x housing interaction (F_interaction_ (8, 152) = 0.7701, p = 0.6296), day x adolescent treatment interaction (F_interaction_ (8, 152) = 0.7319, p = 0.6632), housing x adolescent treatment interaction (F_interaction_ (1, 19) = 1.623, p = 0.2180), or a day x housing x adolescent treatment interaction (F_interaction_ (8, 152) = 1.645, p = 0.1165).

With regards to total fluid, there was a significant effect of housing (Fig 5D, F_housing,_ (1, 19) = 18.28, p = 0.004), by three-way repeated measures ANOVA where single housed total fluid intake was greater than group housed social total fluid intake. There was a significant effect of day (F_day_ (8, 152) = 3.200, p = 0.0022) but not adolescent treatment (F_treatment_ (1, 19) = 0.05834, p = 0.03172). There was a day x housing x adolescent treatment interaction (F_interaction_ (8, 152) = 2.048, p = 0.0444). There were no significant interactions in day x housing (F_interaction_ (8, 152) = 1.454, p = 0.1781), day x adolescent treatment (F_interaction_ (8, 152) = 0.3772 p = 0.9314), or housing x adolescent treatment (F_interaction_ (1, 19) = 0.00647, p = 0.9367). Separate two-way repeated measures ANOVA were run for total fluid intake within each housing condition to tease apart the significant interactions. For group housed social mice, there were no significant main effects of adolescent treatment (F_treatment_ (1, 10) = 0.06171, p = 0.8088) or day (F_day,_ (8, 80) = 1.821, p = 0.0851) or day x treatment interaction (F_interaction_ (8, 80) = 1.822, p = 0.0849. In single housed mice, there was a significant effect of day (F_day_ (8, 72) = 2.368, p = 0.253), but no significant effect of adolescent treatment (F_treatment_ (1, 9) = 0.0031, p = 0.9570), or interaction between treatment and day (F_interaction_ (8, 72) = 0.9268, p = 0.4999).

## Discussion

Social experience is a critical component of adolescent development and the social context in which adolescents drink can affect their susceptibility to problematic drinking later in life. This series of experiments was designed to explore the use of various housing models and slight modifications of traditional 2BC or DID paradigms to measure adolescent ethanol intake during the course of social isolation during this critical developmental period. Since drug seeking and consumption is considered to have a social component [45–47], we originally chose to explore our novel neighbor housing environments [35]. These neighbor cages allow mice to partially interact with two other conspecifics while enabling us to quantify individual ethanol intake of each mouse without expensive monitoring equipment [34]. We then performed a series of studies to investigate the role of social housing (single, neighbor, or group) on adolescent ethanol binge drinking. Under multiple conditions using a limited access DID model, these experiments show that mice that drink alone consumed more ethanol than mice that drink together in groups. As seen in Experiments 2, 3, 4, and 5, the differences in ethanol intake between group housed social and single housed animals are apparent early on in their drinking sessions, and this difference is maintained throughout the adolescent period. The effect size values for these studies were large, suggesting a robust effect of adolescent housing condition on ethanol drinking. This may be suggestive of the effects of increased social stress on the isolated mice and a choice between social interaction or drinking in the group housed mice.

Using our neighbor housing complexes, we first investigated whether ethanol intake and preference were altered in socially isolated mice as compared to mice that had limited physical contact (Fig 1). We had previously found that the neighbor complexes could partially mitigate the emotional, social, and cognitive deficits in post-weaning social isolation [34, 35]. Although ethanol intake increased over the three weeks of 2BC access, drinking in proximity to another mouse, but not in the same physical space, did not affect ethanol intake in males or females. This replicated our prior findings [34]. Removing the partial barriers in Experiment 2 to allow mice to travel between cages did not alter ethanol intake in neighbor housed mice in comparison to single housed or group housed social mice in the DID model. Few studies have explored a housing paradigm similar to the neighbor housing complexes; however, partition housing has been used for drinking studies. Using such a housing paradigm with mesh dividers between adult mice, Van Loo *et al*., 2007 found that living together, but apart, was more stressful than single or group housing based on physiological measures such as heart rate [48]. In adult males, mesh dividers transiently placed between rats during drinking sessions did not affect ethanol intake as compared to single housed rats [49]. Studies by Tomie *et al.* have shown a barrier cage can affect intake in late adolescent (~P49) CD1 mice, but it is sex specific. Females drank increasing amounts of ethanol in the presence of increasing number of cage mates of either sex, but this effect was absent in males [32]. While we expanded our ethanol sessions to include a late adolescent period in Experiment 1, we did not observe significant differences in single versus neighbor housing even in late adolescence. Our lack of differences in ethanol intake between single and neighbor housed adolescents may reflect similar stress-induced drinking in the neighbor housed mice, although this has not been directly assessed. We have previously shown that neighbor housing was beneficial in alleviating the cognitive deficits seen in single housed adolescent males and females; it altered anxiety-like and novelty-seeking behaviors in comparison to isolated and group housed animals [34, 35]. However, the neighbor complexes did not alter ethanol intake or preference in a 2BC model in adolescent or adult male and female C57BL/6J mice as compared to single housed mice [34]. Gene expression changes related to synaptic development in the nucleus accumbens and extracellular matrix, GABA signaling, and protein folding in the prefrontal cortex were altered in adolescent mice that were exposed to the neighbor complexes as compared to single housed mice [34], suggesting that social housing conditions alter adolescent brain development. These differing behaviors could suggest neighbor housing is still a significant enough stressor to induce ethanol intake to that seen in isolated mice [50]. Alternatively, our neighbor complexes could serve as a type of enrichment, especially in the case when the barriers are removed to allow the mice to travel between four connected shoebox cages. Our recent study of gene expression changes in the prefrontal cortex identified an interaction between ethanol drinking and single vs. neighbor housing in a set of immediate early genes that play known roles in activity-dependent plasticity, suggesting that social context in adolescence may alter the basal activity in the prefrontal cortex which interacts with ethanol intake [34]. Environmental enrichment can sometimes, but not always, decrease ethanol intake [51–54]. The presence of additional cotton nestlets as enrichment was sufficient to decrease ethanol intake in isolated mice closer to the observed lower intake of group housed mice [52]. It is possible that adolescent mice are more susceptible to forms of enrichment compared to older animals when enrichment occurs after 8 weeks of age (see review by Simpson and Kelly [55]).

Since we cannot avoid the potential influence of environmental enrichment from the neighbor cages, we tested whether placing mice in individual cages during drinking sessions could be used to measure individual mouse ethanol intake in a group or single housed setting. In Experiment 3, we tested whether drinking in a social setting differed from drinking in isolation and directly compared all three conditions: social isolation and drinking in isolation, group housing and drinking in isolation, and group housing and drinking in a social setting. Group housed social mice that drank together in a cage consumed significantly less ethanol than mice that were single housed or mice that lived communally, but had ethanol access in individual drinking cages. Separating group housed mice into individual drinking cages did not robustly change ethanol intake from intake in single housed mice, suggesting that the stress of social separation may also increase drinking [56]; however, this has not been directly tested. Our daily ~6-hour separation during the drinking sessions may also be enough of a social stress to increase ethanol intake. Additional assessments of social stress during these repeated periods of separation would be needed to evaluate such changes in the potential stress-induced ethanol intake.

Based on Experiments 1–3, we conclude that drinking in the presence of other mice decreases drinking, but not when intermittent separation or limited contact occurs during the drinking sessions. We hypothesize that the separation into individual cages for drinking sessions, or prolonged partial contact in adolescence, may act as a stressor in Experiments 1 and 3. Alternatively, having other mice present during a drinking session could be a distraction, or a differently motivated choice [57]. In a model using saccharin-enriched ethanol in a 2BC intermittent access model, rats isolated during adolescence drank more ethanol than group housed animals experiencing intermittent isolation during the adolescent drinking sessions [40]. This is somewhat contrary to our findings with 2BC in Experiment 1 showing social isolation versus neighbor housing, or when group housed mice drank in isolated cages for DID in Experiment 3. The differences may be explained by a number of factors. Their study was conducted in rats, used sweetened ethanol, and food-restricted the adolescent rats during their drinking sessions. Thus, the rats could have been differently motivated to consume ethanol. In one study design, rats isolated at weaning were pair housed on P43 and at the start of adulthood (~P60), all animals were isolated for the drinking study [58]. Post weaning isolation increased drinking behaviors in rats compared to their pair housed counterparts. Animals with a history of isolation during adolescence drank more and had higher preference for ethanol [58]. While adolescent social isolation tends to increase voluntary ethanol consumption, this is not always the case. Rivera-Irizarry *et al.* single housed male and female mice at weaning for six weeks and found no differences in ethanol DID intake in adulthood. They did note that these mice displayed a hyper-social phenotype, further supporting the idea that isolation may affect motivation [31].

One of the pressing questions in this study was if the social drinkers were drinking less due to there being only one bottle for four mice in a cage. Irrespective of the number of bottles available, socially isolated male mice drank significantly more than group housed social drinkers (Experiment. 4). Szumlinski *et al*. 2019 reported a similar finding in which the number of bottles and concentrations of ethanol available to adult mice was varied. While increasing the number of available ethanol concentrations tended to increase total ethanol intake, there were no differences between the groups in both sexes [56]. Similar increases in total ethanol intake with increased number of tubes was observed in two other studies; the more tubes present, the higher the relative ethanol intake [59, 60]. A recent study tested single versus group housed adult C57BL/6J males to assess intake via HM2 cages, which employ an RFID chip to record individual ethanol intake information [61]. The study found that socially housed males drank significantly more ethanol than socially isolated males, a trend opposite of what we report here using adolescents. The importance of social play in adolescence may underlie this difference and may be sex-dependent. In rats, single housed adolescent females consumed more ethanol than group housed females, while single housed adolescent males and adult male and female rats consumed more sweetened ethanol under social circumstances than when individually housed [62]. Pair-housed male rats also had a higher ethanol intake and preference that decreased over time as compared to single housed rats; females were unaffected [63]. Highly playful adolescent rats were more prone to alcohol intake, yet showed greater control over alcohol seeking [64]. Together, these studies suggest a critical role of social play in the development of emotion and cognitive control and that social context modulates reward seeking. Future studies are needed that can directly assess the interaction of social play, social anxiety and binge ethanol drinking behavior in adolescent rodents.

Other 2BC studies have shown that adolescent social isolation increases ethanol drinking in adulthood compared to their group housed counterparts (see review [65]). We also report that a history of binge ethanol increased adult intake in a 2BC model (Fig 5B). However, this only reached significance in mice that were group housed social drinkers. Group housed social ethanol mice drank more than group housed social water males, particularly during the first week of access in adulthood (Fig 5B). It is possible that ethanol intake in single housed mice has reached a ceiling and prior exposure in adolescence cannot further increase ethanol intake in the 2BC model in adulthood. Our earlier studies in single versus neighbor housed mice found that an adolescent history of ethanol did not alter adult drinking in either housing condition [34]. Following non-contingent ethanol exposure in adolescent rats (0 or 2g/kg i.p. from PND 30–43), neither prior exposure to ethanol, nor single or group (with or without enrichment) housing altered ethanol intake in a 2BC model in adulthood [54]. The authors acknowledged that one reason could be that group housed rats were single housed for 2BC, which could affect drinking behavior [54]. Our current findings are in line with several previous studies from other groups showing adolescence is a critical period for ethanol induced risk for increased adult intake, but this phenomenon may be altered by stress [65].

There are several limitations to the current set of exploratory studies. These studies investigated ethanol drinking behavior in adolescent mice while drinking alone versus drinking together using a series of housing conditions with a simple, inexpensive design. We found that mice that drink alone consistently consumed more ethanol than mice that drink together in groups, but we do not know why. More sophisticated tracking of individual mouse social and affective behaviors during ethanol drinking bouts is needed to dissect the relationship between sociability in mice and ethanol drinking behavior. The social structure of the mice in the current study is also unknown and should be included in further investigatory studies. Our earlier investigations into the use of the neighbor housing arenas have shown that neighbor housing can mitigate some of the social, affective and cognitive changes seen in post-weaning social isolation [34, 35]. Neighbor housed mice are more behaviorally similar to group housed mice, than single housed mice and showed fewer deficits in the novel object recognition task, lower anxiety-like behavior in the light-dark box and increased social interactions [34]. However, ethanol drinking behavior did not differ between single and neighbor housed mice, suggesting that broader changes and interactions in the developmental trajectory may be underlying these behavioral differences. Indeed, our initial transcriptomics studies in the prefrontal cortex and nucleus accumbens suggest that housing conditions (social isolation and/or enrichment in the neighbor cages) during this critical period of adolescence can alter the trajectory of cortical development, which is likely underlying some of these behavioral differences [34, 35]. As social interaction and social play are critical for the development of emotion and cognitive control in adolescence, further studies need to be performed to investigate the structural and neurochemical changes that drive developmental stunting associated with single housing. All mice in these studies were obtained from Jackson Laboratories and shipped as adolescents. Shipping can certainly alter sex-specific behaviors in mice and increase stress reactivity [66]. Future studies should investigate such additional stressors and consider in-house breeding strategies to reduce such confounds.

Importantly, most of the current studies were performed in males; female rodents routinely consume more ethanol than males [65]. The primary objective of this set of experiments was to examine the effects of housing on ethanol intake in adolescence and adulthood. Our *a priori* hypothesis that males and females drink differently is supported by the literature [52, 67, 68]. Future studies will explore how adolescent isolation and binge drinking affect adult intake in females. As far fewer voluntary drinking studies have been published using females, it is difficult to speculate whether estrous onset and cycling would affect intake in our particular study. One study tracked estrus in adults and noted it did not affect drinking behaviors using a DID procedure [69]. The study also compared this to ovariectomized females’ ethanol intake and found treatment with estradiol prior to the session increased intake compared to untreated ovariectomized females [69]. The social behaviors of males and females differ greatly as well – males tend to engage in more “play fighting” behaviors, exhibit higher levels of aggression, and form dominance hierarchies by the time they reach adulthood; this behavior is not characteristic in females and shows heightened displays in adolescents versus adults [70]. While this type of play fighting behavior is not overt during adolescence, aggression may become a factor following puberty and may lead to sex differences in response to social isolation and in ethanol preference during binge drinking.

Adolescence is a period of extensive neurobiological development, particularly in the prefrontal cortex. Inputs from other brain regions such as the amygdala, hippocampus, and within the prefrontal cortex itself help to mature and refine its circuitry [71, 72]. Adolescent social isolation and binge ethanol alter glutamatergic activity, long-term potentiation, and dendritic spine density and morphology in multiple brain regions (see review by Lodha *et al.* 2022 [65]). Though the specific mechanisms underlying these changes remain unknown, impaired GABA maturation or synaptic development may contribute to the cognitive, social, and anxiety-like deficits observed after adolescent isolation or binge ethanol. Further dissection of the cell populations in the prefrontal cortex activated by novelty and drug seeking behaviors will shed some light on how these changes occur during adolescence and how drug exposure or isolation alters these populations.
